# Influence of advanced shoe technology on the top 100 annual performances in men’s marathon from 2015 to 2019

**DOI:** 10.1038/s41598-021-01807-0

**Published:** 2021-11-17

**Authors:** Víctor Rodrigo-Carranza, Fernando González-Mohíno, Jesús Santos del Cerro, Jordan Santos-Concejero, José María González-Ravé

**Affiliations:** 1 Sport Training Laboratory, Faculty of Sport Sciences, University of Castilla-La Mancha, Avenida Carlos III S/N, 45071 Toledo, Spain; 2 Department of Statistics, University of Castilla-La Mancha, Toledo, Spain; 3 Facultad de Ciencias de la Vida y de la Naturaleza, Universidad Nebrija, Madrid, Spain; 4 Department of Physical Education and Sport, University of the Basque Country UPV/EHU, Vitoria‑Gasteiz, Spain

**Keywords:** Biological techniques, Physiology

## Abstract

The NIKE Vaporfly shoe was introduced in May 2017 as part of the original #Breaking2 Project (an event aimed to run the first marathon under 2 h). This new advanced shoe technology (NAST) changed the footwear design conception. The aim of this study was (i) to analyse the effect of NAST in men’s marathon performance, (ii) to analyse whether the changes in the environmental constraints (temperature and wind) and orography of the marathons, age and birthplace of the runners has changed from 2015 to 2019 and (iii) to analyse the impact of NAST on the historical 50 best performances. Data from top-100 men's marathon performances were collected in that timeframe. The shoes used by the athletes were identified (in 91.8% of the cases) by publicly available photographs. External and environmental conditions of each marathon and age and birthplace of the runners were also analysed. Marathon performances improved from 2017 onwards between 0.75 and 1.50% compared to 2015 and 2016 (p < 0.05). In addition, the improvement was greater in the upper deciles than in the lower ones (p < 0.001). Runners wearing NAST ran ~ 1% faster in marathon compared to runners that did not use it (p < 0.001). When conducting an individual analysis of athletes who ran with and without NAST, 72.5% of the athletes who completed a marathon wearing NAST improved their performance by 0.68% (p < 0.01). External and environmental conditions, age or birthplace of runners seems not to have influenced this performance improvement. NAST has had a clear impact on marathon performance unchanged in the environmental constraints (temperature and wind), orography, age, and birthplace of the runners but with differences between venues.

## Introduction

The sub-2 h barrier in the marathon has attracted attention from sports scientists and footwear manufacturers over the last decade^1,2^. Previous research has proposed marathon performance prediction models that take into account physiological determinants (i.e. maximum oxygen uptake [VO_2max_], lactate threshold [LT] and running economy [RE] mainly)^3,4^. Currently, elite marathon runners have relatively high VO_2max_ values (ranging from 70 to 85 ml·kg^−1^·min^−1^), although these values are similar to those reported in the sixties^5,6^. Therefore, other factors apart from improved cardiac output and arteriovenous oxygen difference must be considered. In this regard, RE and the LT speed appear to be the most important variables when aiming for elite running performance in the marathon^7^. The nature of the physical work involved in this event requires to run without accumulating lactate (metabolic steady-state), using fat as a fuel at high work rates and running at race pace with low energy cost of running (good RE)^8^. Typical values of RE and speed of LT in elite distance runners have been reported previously^6,9^, such as 187 ml/min/kg and a speed of LT of 90% of VO_2peak_ of elite east Africans.

A statistical approach proposed by Angus^10^suggested that the expected first sub–2 h marathon would have to wait until May 2032, and other researchers, by comparing the gender gap between different running events, concluded that an imminent sub-2 h was not likely^11^. However, the last October 2019, the Kenyan champion Eliud Kipchoge ran the first sub-2 h marathon in Vienna in an unofficial event (not homologated as official World Record by the Technical rules of *World Athletics*) with favourable orography and the assistance of drafting strategies and pacemakers. Recently, it has been shown that the event profile^12^, the environmental temperature^13^ and other external conditions such as unconventional drafting strategies could affect the athletes' race pace^14^.

Moreover, in May 2017, the #Breaking2 project by NIKE introduced a new advanced shoes technology (NAST), the NIKE Zoom Vaporfly Elite. This type of footwear is characterised by its lightness, a midsole composed of a novel compliant and resilient ZoomX foam (PEBAX), which provides more energy return and a carbon fibre plate inserted in the midsole with the goal of improving the longitudinal bending stiffness^15–17^. The stiff curved plate and the resulting “teeter-totter” effect of the NIKE Vaporfly and other similar NAST models from other brands has contributed to improve road running performance^18,19^. RE improvements when compared to conventional shoes range between 1 and 6%^15,16^ and the benefits in the marathon event have even been compared to the performance benefit of erythropoietin doping^20,21^. As a result, the *World Athletics* has been compelled to control this revolution of the NAST models by promoting new rules on shoe technology for long-distance races^22–24^.

Interestingly, until 2014 (the last world record before the appearance of this NAST models revolution was 2:02:57 by Dennis Kimetto, Berlin Marathon, 28 September 2014), the marathon world records only improved by 3.06% in the previous 25 years^25^. However, just after this new shoe technology was launched, the world records improved by 1.06% and 1.00% for men and women in a very short period of time. Furthermore, half of the 50 best times in history have been achieved since the creation of this type of footwear. Thus, it seems that recent technological advances are critical factors affecting running performance related variables (RE and LT speed) that can explain most of the recent improvements in the marathon.

Therefore, the aims of this study were (1) to analyse to what extent the NAST have influenced the revolution of marathon performances in recent times, (2) to analyse whether the changes in the environmental constraints (temperature and wind), orography, age, and birthplace of the runners have changed in the years analysed, and (3) to analyse the impact of NAST on the historical 50 best times. We hypothesised that the emergence NAST has influenced significantly marathon running performances at the top level, and the environmental constraints, orography, age, and birthplace of the runners did not differ between the years analysed, and therefore, they have not influenced in the performance improvements.

## Methods

The top-100 performances in men’s marathon from 2015 to 2019 were gathered from a publicly accessible website (https://www.worldathletics.org, accessed 20 November 2020), with a total of 500 entries. 40 marathon runners out of a population of 217 athletes completed a marathon without and with NAST in the years analysed. Therefore, 80 entries corresponded to the same athletes without and with NAST in the years evaluated In addition, the top 50 performances in history were also analysed, with the aim of analysing the impact of NAST on the historical best times. This study involved the analysis of publicly available data so that individual informed consent was not necessary. The Nebrija University Research Ethics Committee approved the study (UNNE-2020-010), and it was performed according to the declaration of Helsinki. Only men were selected as it was impossible to detect footwear for most of the top 100 women between 2015 and 2019. The years 2015 and 2016 were evaluated to analyse the impact of the NAST footwear that started in 2017.

Total entries were selected instead of the best performance of each athlete as in the study of Marc et al.^26^, because the same athlete could wear (or not) NAST depending on the race in the same year, being a bias for NAST.

Two experimented researchers (VRC, FGM) analysed the media content (official photos, and footage of athletes in competition of the web and social networks of the event and runners) of each runner to identify the shoe worn by runners in each race. Although the NIKE Vaporfly were introduced in May 2017, prototypes of this model were detected at the 2017 spring marathons and at the 2016 Olympic Games and were therefore controlled. Results were compared and discrepancies were resolved by consensus or by consulting the senior author (JMGR). We identified the footwear worn in 456 of 500 (91.8%) marathon runners in our dataset. The prevalence of NAST in the years analysed was 156 cases between 2015 and 2019, while the incidence by years was 0, 4, 21, 59, 72 for years 2015, 2016, 2017, 2018 and 2019, respectively. The 99.60% of the footwear identified corresponds to the NIKE Vaporfly model. The external and environmental conditions of each marathon were obtained to make a more realistic comparison of the effect of the NAST. Temperature and wind for the day of each race were collected from Weather Spark (https://es.weatherspark.com/), and the marathon characteristics from the social media platform Strava (average altitude, increase and decrease in meters), as done elsewhere^27,28^. Thus, each entry in the database included at least: official final time, final position, date, place of the marathon, if the runner used a NAST or not, age of the runner and environment characteristics of the marathon (temperature, altitude, and wind).

### Statistical analysis

One-way analysis of variance (ANOVA) with subsequent Tukey post-hoc test was used to compared data across years and the use or non-use of NAST. The top 100 performances were categorised into performance deciles and two-way repeated ANOVA (decile × year) was used to compare the differences across years. A *t*-test for unpaired groups was used to compare the use or non-use of NAST. We calculated the effect size using the partial eta squared (ŋ2). Values of 0.01, 0.06 and above 0.15 were considered as small, medium, and large, respectively^29^. In addition, we calculated the effect size using Cohen’s *d* for post-hoc analysis. Values of 0.49, 0.5–0.79, and ≥ 0.80 were considered as small, medium, and large, respectively^29^. Finally, to assess the relationship between the use of NAST and performance improvement over the years, a Pearson’s product-moment correlation coefficient (r) was used. All analyses were performed with the software R. Figures has been performed with Graph Pad Prism software (version 8.0 for Mac). Significance for all analyses was set at p < 0.05.

## Results

### Performance

**Figure 1:**
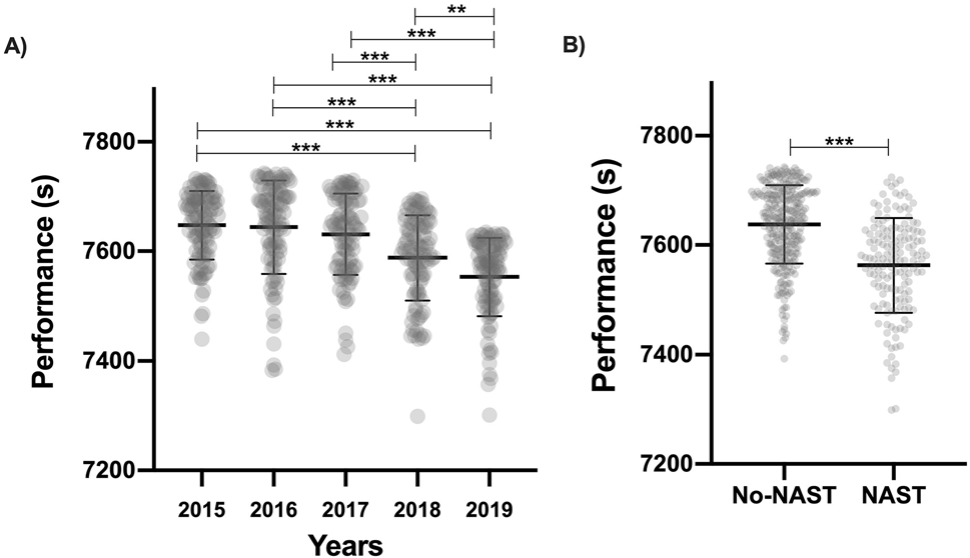
Distribution of the individual and average finishing times from the top-100 performances per year from 2015 to 2019 **(A)**. Distribution of the individual performances and average times from runners with and without NAST **(B)**. **p ≤ 0.01; ***p ≤ 0.001.

Figure 1A shows the changes in men’s marathon performances in the years analysed. There were significant differences across the years (F = 29.91; p < 0.001; ŋ^2^ = 0.19). Marathon performances improved by 0.88% and 1.50% from 2015 (7647 ± 62.75 s) to 2018 and 2019 (7588 ± 77.74 and 7553 ± 71.15 s) (p < 0.001; Cohen’s d = 0.88, 1.47), by 0.74% and 1.20% from 2016 (7644 ± 85.48 s) to 2018 and 2019 (p < 0.001; Cohen’s d = 0.68, 1.15), by 0.57% and 1.13% from 2017 (7631 ± 74.47 s) to 2018 and 2019 (p < 0.001; Cohen’s d = 0.56, 1.07), and by 0.47% from 2018 to 2019 (p < 0.05). However, the change in performance between 2015 and 2017 remained between 0.14 and 0.21% (ns).

When the use of NAST was assessed, there were significant differences in men’s marathon performance as well (T = 9.97; p < 0.001; Cohen’s d = 0.98). Runners wearing NAST had an average performance of 7563 ± 86.1 s compared to 7637 ± 72.5 s of runners not using NAST (Fig. 1B).

**Figure 2:**
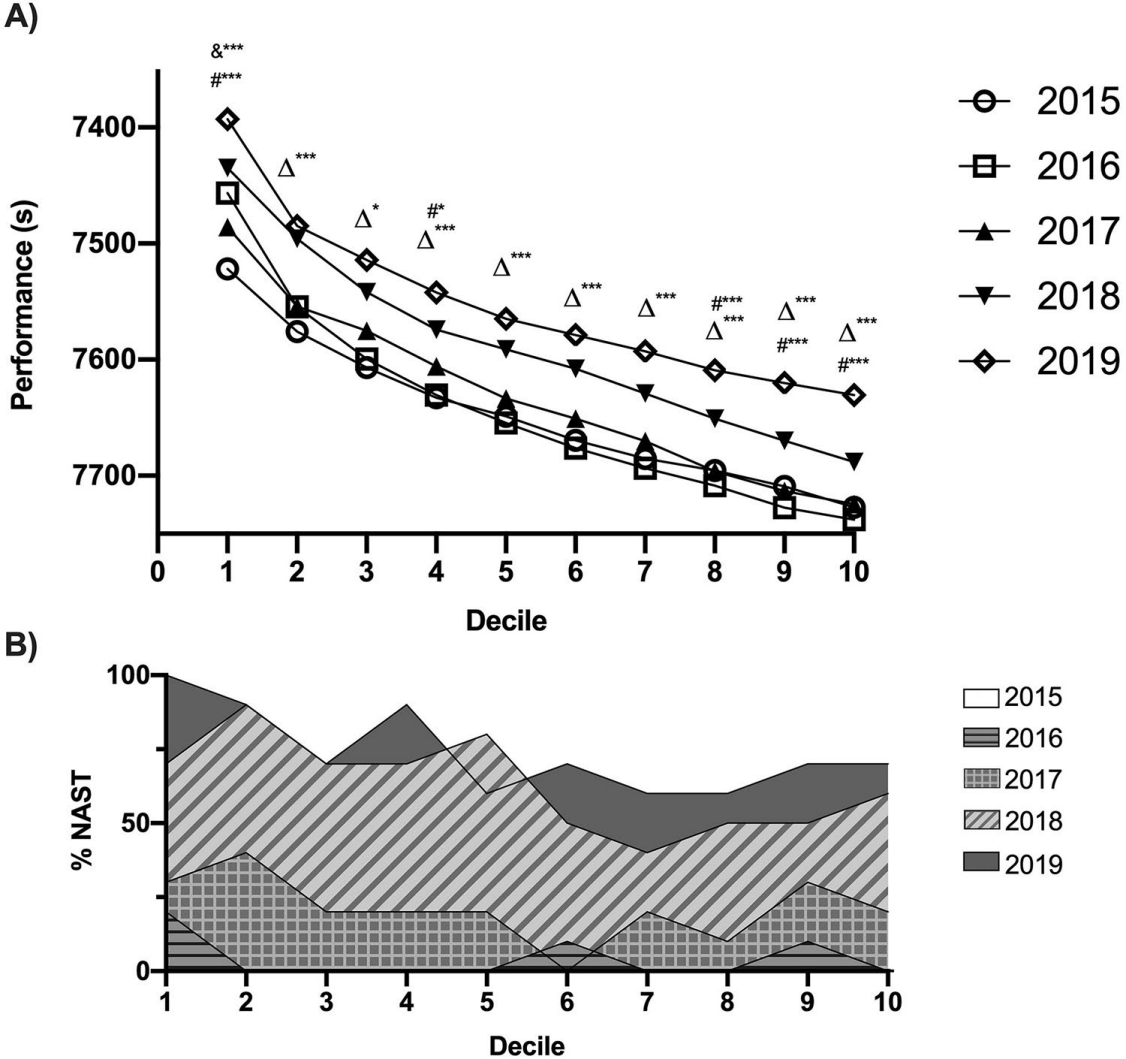
Analysis of average finishing times per decile from 2015 to 2019. & Mean difference between all years; Δ differences between 2015, 2016 and 2017 with 2018 and 2019; # differences between 2018 and 2019. *p ≤ 0.05, ***p ≤ 0.001.

The mean performance of the top 100 marathon runners has continually improved in all deciles (Fig. 2). However, the increase has been greater in the upper deciles than in the lower ones (p < 0.001). For example, when comparing the times in 2015 and 2016 (pre-NAST) decile 1 (best 10% of performances) has improved by 1.74% (p < 0.001) and 2.24% (p < 0.001), while the decile 10 by 1.27% (p < 0.001) and 1.41% (p < 0.001) compared to 2019, respectively.

There was a high correlation between performance improvements by year and the number of runners using NAST (r = 0.96; p = 0.01), which means that as the number of runners wearing NAST each year since 2017 increases, the greater the average performance improvement is. The percentage of runners wearing NAST per year was of 0%, 4%, 21%, 63% and 84%, for 2015, 2016, 2017, 2018 and 2019, respectively. The performance improvement regarding the previous year was of 0 s, 3 s, 13 s, 43 s and 35 s, for 2015, 2016, 2017, 2018 and 2019, respectively. Considering the impact of the NAST on the best times in history, 23 of the 50 best times in history (accessed 20 November 2020) (46%), have been achieved with NAST, with twenty-one of them being achieved since 2018 (42%).

### Analysis of cases of repeated performances

From the total number of athletes evaluated, 40 athletes completed a marathon without and with NAST in the years evaluated. 29 of the 40 athletes (72.50%) improved their performance with this type of footwear, while the remaining 27.50% did not benefit. Running in NAST improved performance by 0.68% (p = 0.005) for the whole group of athletes tested (from 7632.45 ± 68.56 s to 7580.2 ± 78.73 s, for runners without and with NAST, respectively).

### Age

We found no age effect in the years analysed (28.55 ± 4.10, 27.97 ± 4.27, 27.36 ± 3.66, and 28.36 ± 4.03 years, for 2015, 2016, 2017, 2018 and 2019, respectively) (Table 1).

**Table 1:** Results of the age, environment and marathon characteristics across the years analyzed.

	Years					One-way ANOVA	
	2015	2016	2017	2018	2019	*p*	Effect size (η^2^)
Age (years)	28.55 ± 4.10 (20–42)	27.97 ± 4.26 (20–43)	27.36 ± 3.65 (21–35)	28.36 ± 4.03 (21–42)	28.03 ± 4.11 (21–38)	0.28	0.01
**Environment**							
Temperature (ºC)	12.28 ± 4.38^⍏^ (6–25)	13.64 ± 4.38 (8–25)	13.23 ± 3.47 (6–24)	13.66 ± 4.68 (5–22)	14.07 ± 4.34 (5–24)	0.04	0.02
Wind (km/h)	9.65 ± 5.14^#⍏^ (1–27)	9.63 ± 4.46^#⍏^ (3–22)	10.41 ± 6.81 (4–32)	12.20 ± 6.61 (4–31)	12.39 ± 6.74 (4–28)	< .001	0.03
**Marathon characteristics**							
Average altitude (m)	60.80 ± 94.78 (0–466)	74.89 ± 230.03 (0–1590)	41.73 ± 74.61 (0–399)	64.32 ± 92.82 (0–399)	61.92 ± 100.05 (0–399)	0.50	0.00
Ascend (m)	145.60 ± 91.38 (14–349)	146.06 ± 85.30 (14–327)	148.26 ± 90.43 (14–327)	122.65 ± 81.91 (14–349)	130.18 ± 78.79 (1–349)	0.21	0.01
Descend (m)	154.74 ± 95.59 (14–347)	151.49 ± 90.87 (14–328)	153.78 ± 95.77 (14–328)	126.34 ± 89.09 (14–347)	133.15 ± 82.51 (4–347)	0.14	0.02

Data are presented as mean ± standard deviation and range (minimum–maximum).

Post hoc analysis: ^#^Different from 2018 (p < 0.05); ^⍏^Different from 2019 (p < 0.05).

### Environment

The average temperature of the marathons remained the same from 2016 to 2019. We only found differences between 2015 and 2019 (p < 0.05). The wind in the marathons analysed increased in 2019 compared to 2015 and 2016 (p < 0.05) (Table 1).

### Marathon characteristics

**Figure 3:**
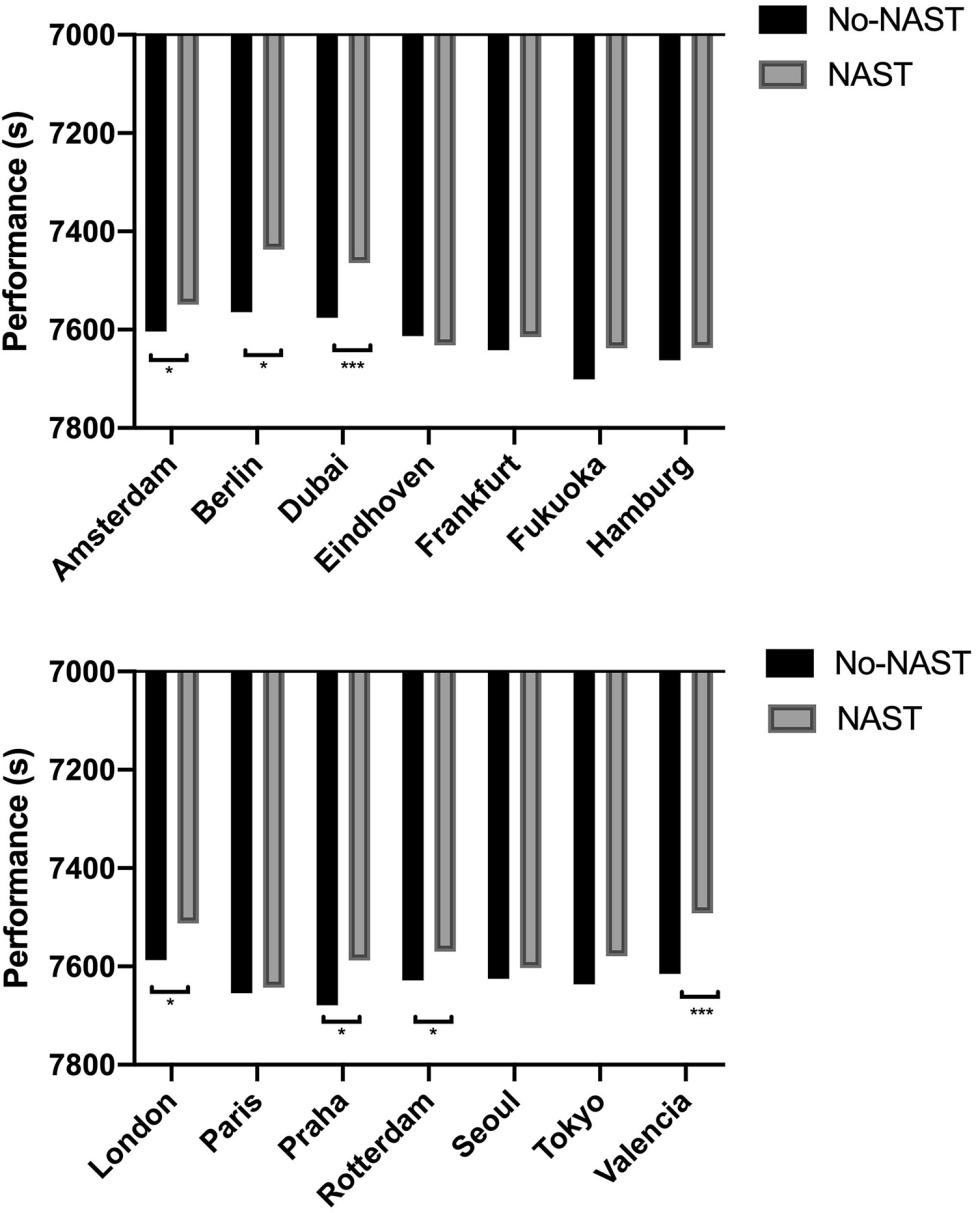
Average finishing times in different venues of athletes with and without LBS. * p ≤ 0.05, *** p ≤ 0.001. Participants in each venue were: 44 in Amsterdam, 22 in Berlin, 35 in Dubai, 18 in Eindhoven, 20 in Frankfurt, 10 in Fukuoka, 16 in Hamburg, 27 in London, 27 in Paris, 13 in Praha, 25 in Rotterdam, 35 in Seoul, 32 in Tokyo, 46 in Valencia.

We found no effect for any of the marathon characteristics evaluated. The average altitude, metres ascended, and metres descended remained unchanged across the years (Table 1). For the venues that were repeated during the years evaluated, the venues where performance was improved significantly with this type of footwear were Amsterdam, Berlin, Dubai, London, Prague, Rotterdam and Valencia (Fig. 3).

### Birthplace

The distribution of athletes according to their birthplace where athletes were born did not change in the years analysed, with 86.4% of the athletes coming from Africa, 0.40% from America, 3.60% from Asia and 2.4% from Europe.

## Discussion

Our study evaluated the effect of wearing NAST from 2015 onwards. The main finding of this study was that top 100 men’s marathon performances have improved over the years from 2015 to 2019, especially after 2017 coinciding with the launch of the NAST. The second finding of this study was that the environmental constraints, orography, age, and birthplace of the runners did not change. Therefore, the NAST footwear seems to have had an influence on the performance improvement in this event.

The first analysis of the reasons that may explain why marathon performances improved dramatically from 2017 onwards was to compare runners wearing NAST with those who did not. Out of the 500 performances analysed and taking into account that the footwear brand and model was checked/confirmed for 91.8% of the sample, the 34% of the performances were achieved by runners using NAST. The marathon performance of the runners with this type of footwear was 0.98% better than those of runners without it. The year-to-year improvement marathon performances between 2015 and 2017 was only of 0.14–0.21%. However, from 2017 onwards the performance improved between 0.75 and 1.50% compared to 2015 and 2016. Therefore, the relationship between the performance improvements and the appearance of this type of footwear is clear. In addition, there was a strong correlation between number of runners using NAST per year and marathon performance improvements (r = 0.96), means that the greater number of runners using NAST per year, the better was the average improvement of the top-100 marathon runners.

When analysing the evolution of marathon performances, Joyner et al.^1^ estimated that if the trend existing since 1960 had continued, with a performance improvement of 10–20 s per year, a sub-2 h would have been possible by 2021–2022. However, when analysing the marathon performances just before the release of the first NIKE Vaporfly model (2015 and 2016) the improvement per year was of only of 3 s in the average of the 100 best performances. These findings agree with previous research that showed how just before the release of this shoe model in Monza 2017, the marathon performances were stable or even were slower^11,30^. Tucker and Santos-Concejero^11^ even suggested that achieving the 4% improvement needed to break the sub 2 h marathon would take longer than expected previously, because there were no potential changes in training, and the doping was more controlled than before. However, when comparing the average performances between 2017 and 2018, and 2018 with 2019, the improvement increased to 43 s and 35 s, respectively.

When analysing the evolution of performance by dividing the sample into level deciles (Fig. 2), the fastest athletes improved the time performed in a marathon, in contrast to previous assessment studies on marathon performances where all deciles varied in the same way^26^. The reason why it benefits more to faster athletes could be due to the involvement of the metatarsophalangeal (MTP) joint with this type of footwear, which improvements at higher running speeds, and the instantaneous stiffness which fluctuates throughout the stance phase, suggesting that the foot-shoe complex stiffness is time dependent^31^ and explaining by the reduced energy loss of the MTP joint^32^. As RE improves to a greater degree as speed increases^16,17,33^, high level athletes may benefit more than recreational athletes when using this type of footwear.

When conducting an individual analysis of athletes who ran with and without NAST, most of them (72.5%) improved their performance by 0.68% with this type of footwear. As suggested above, not all athletes could benefit from the use of this type of footwear^15^. A possible explanation could be that there is no linear relationship between the longitudinal bending stiffness (LBS) of the midsole and RE. Roy and Stefanyshyn^34^ analysed the influence of the footwear with increased of LBS and they found a U-shape relationship between LBS and RE, suggesting that there is an optimal LBS in terms of improved energy cost of running. These authors found a negative correlation between subjects’ mass and variations in RE, suggesting that the optimal LBS may be dependent on the runner’s mass. In addition, they suggested that may be a limited range of running footwear LBS suitable to improve RE for each athlete. Moreover, previous studies have shown that greater mass at a distal point, such as in shoes, impairs performance, especially at higher intensities^35^. Thus, we could assume that the light weight also characteristic of this type of footwear compared to conventional shoes is other factor related to the improvement of marathon performance.

Besides, there are important external variables such as marathon characteristics and environmental factors that must be discussed. It has been suggested that characteristics of the race, such as the course and the environmental temperature, could affect the athletes' race pace^13^. Most studies have been done on specific marathons^36–38^, which may have influenced the results and make it difficult to extrapolate to other situations. In fact, there is a controversy about how the profile of the races would affect performance. Haney and Mercer^13^ found no differences between the Las Vegas and San Diego marathons despite the different elevation profiles of the two races. However, a recent study suggests that the characteristics of the marathon affect more the running pace of recreational athletes than high-level athletes^39^.

According to our analysis, our results showed no differences between the characteristics of the different marathons, so the circuits have not affected the average performances of the years analysed. However, not in all venues athletes have run faster with NAST, which could mean that there are more convenient circuits for running with this type of footwear. The circuits where runners have run faster are, in the following order: Amsterdam, Berlin, Dubai, London, Prague, Rotterdam and Valencia. Most of these circuits are commonly recognised as fast by runners. It is known that allowing a relatively even pace with few changes of direction and minimum elevations and a more controlled energy demands may led to improvements in marathon performance, although specific research is needed with this type of footwear^40^.

Thermoregulation is another important consideration and therefore, environmental factors including temperature, radiant heat and wind speed may significantly influence marathon performance^41^. It has been proposed that endurance athletes perform better in colder environments^42^. In fact, heat can cause significant alterations of cardiovascular, metabolic, neuromuscular and thermoregulatory functions in long-distance races such as the marathon, besides, hyperthermia appears to be a key factor limiting exercise performance in hot conditions^43,44^. A study of the Stockholm Marathon^45^ and a large epidemiological study of all participants in the Boston, Chicago, New York, Paris, London and Berlin marathons from 2001 to 2010 along with a recent study conducted at the New York City Marathon^46^ confirmed this trend and showed that ambient temperature is influential^47^.

In our study, we found differences between the temperature of 2015 (~ 12 ºC) and 2019 (~ 14 ºC), but in all years the temperature remained between 12 and 14 ºC, which is slightly higher than the temperature considered ideal for the marathon^12,41^. Definitely, the performance improvement from 2017 onwards compared to the previous years (2015 and 2016) is not a consequence of a better weather conditions or advantageous course profiles of the races.

Historically, the best marathon performances have taken place in April and October^26^, for example in London, Rotterdam, Paris, Berlin, Chicago and Amsterdam. The top 100 annual performances have historically been achieved in 31 races^26^. However, our study shows a trend in the reduction in the number of races where the top-100 marathon performances are made per year, being 30, 32, 26, 26 and 26 from the years 2015 to 2019. Therefore, it seems that less marathon venues monopolise the best marathon performances since the release of the NAST. However, we cannot confirm that this is due to the effect of this type of footwear or that simply that athletes prefer to run on faster courses.

The age of the athletes remained unchanged during the years evaluated. The average age of the top 100 times each year was very close to the peak performance age for the marathon^37,48^ so it seems that NAST do not change the peak performance age for the marathon.

The demographics of athletes also remain unchanged, with a clear African predominance (mainly from Kenya and Ethiopia) accounting for about 95% of all athletes, in agreement with previous research^26^. Several studies have tried to explain the success of Africans, based mainly on biomechanical and physiological causes^49,50^ but it seems that the use of the shoes analysed does not cause a change in the trend of the origin of the athletes.

We are indeed conscious about the complexity of controlling all dependent variables which may affect the final performance in a marathon. However, top-level athletes generally tend to remain stable over time in the way they train through the season and obviously, they are aware of the of their physiological limits^6^.

Therefore, this difference cannot be explained by the fact that the fastest runners choose to use this footwear, because they use it in more favourable races or because they use it after they have trained more. In a race between two athletes of the same ability, the athlete wearing NAST will have a significant advantage.

## Limitations

The main limitation of this study was the research design. We completed a retrospective observational study due the accessibility of an official database such as worldathletics.org. The study included the analysis of publicly available data as an advantage, because the alternative design (a crossover experimental design) would have implied a smaller sample size. In addition. the analysis of the official photos and footage of athletes in competition could contain some errors, although we believe that including two experienced researchers in the team allowed us to minimise the identification mistakes.

## Conclusion

We conclude that NAST, as those released after the NIKE Vaporfly characterised by its lightness, a midsole which provides more energy return, a stiff curved plate inserted in the midsole with the goal of improving the LBS and the resulting “teeter-totter” effect has a clear impact in marathon performance unchanged in the environmental constraints (temperature and wind), orography, age and birthplace of the runners but with differences between venues. These improvements could be due to changes in RE as shown in previous studies with this type of footwear. As a practical application, an elite athlete who has a 2:08:00 marathon time could benefit from around 54 s running in a suitable venue with this type of footwear. Future studies should examine the effect of external conditions such as weather on performance with NAST, i.e. whether better performances are achieved with NAST on rainy days or hot days compared to not using NAST.
